# Enhancement of Corrosion Resistance Properties of Electrodeposited Ni/nano-TiC Composite Layers

**DOI:** 10.3390/ijms23116069

**Published:** 2022-05-28

**Authors:** Nicoleta Bogatu, Lidia Benea, Elena Roxana Axente, Jean Pierre Celis

**Affiliations:** 1 Competences Center: Interfaces-Tribocorrosion-Electrochemical Systems, Dunarea de Jos University of Galati, 47 Domnească Street, RO-800008 Galati, Romania; 2 Faculty of Engineering, Dunarea de Jos University of Galati, 47 Domneasca Street, RO-800008 Galati, Romania; nicoleta.simionescu@ugal.ro; 3 Faculty of Medicine and Pharmacy, 35 Al. I. Cuza Street, RO-800010 Galati, Romania; 4 Department of Metallurgy and Materials Engineering, Katholieke Universiteit Leuven, Kasteelpark Arenberg 44, B-3001 Leuven, Belgium; jean-pierre.celis@kuleuven.be

**Keywords:** composite layer, nano-TiC dispersed particles, nickel matrix, corrosion resistance, electrodeposition, XRD

## Abstract

This paper presents novel results on the effects of the dispersion of titanium carbide nanoparticles (50 nm mean diameter) into a nickel-plating electrolyte on the corrosion behavior of the nanocomposite layers obtained. The Ni/nano-TiC layers are compared with pure nickel layers obtained at the same electrodeposition parameters with 60 mA·cm^−2^ current density and 10 min deposition time. The comparative corrosion performances are investigated using a three-electrode electrochemical cell in a solution (mixed boric acid with lithium hydroxide), which simulates the primary water circuit of pressurized water reactors (PWRs). Open circuit potential measurement and electrochemical impedance spectroscopy were employed as the electrochemical methods, using an electrochemical workstation connected to an electrochemical cell, as well as a PC with software to drive the experimental work. The results clearly revealed enhanced corrosion properties for the Ni/nano-TiC hybrid layers as compared to the pure Ni layers. The significantly improved corrosion behavior can be attributed to the TiC nanoparticles embedded into the Ni matrix, which have the effect of insulating centers at the composite layer/corrosive solution interface.

## 1. Introduction

Nanocomposites are composites in which the dispersed phase has dimensions in the order of nanometers (1–100 nm) [1]. These are high-performance materials for the 21st century and they possess unusual combinations of unique properties and design possibilities that are not found in conventional materials [1]. With an estimated annual growth rate of about 25%, their potential is so great that they can be used in many areas, ranging from packaging [2] to biomedical applications [3]. Nanocomposite applications offer new technologies and opportunities in the nuclear [4,5,6,7], aerospace [8], automotive [9], electronics [10] and biotechnology [11] sectors, making them a niche field in the development of micro-enterprises internationally [8,9,10,11].

In obtaining nanocomposite materials, scientists and engineers have ingeniously combined different metals, ceramics and polymers to produce a new generation of advanced materials with improved properties. Most nanocomposites are designed to improve the mechanical properties of conventional materials [4,5,6,7]. The technology used to obtain nanocomposite layers is the basis of nanocomposites’ many applications, and currently there are a large number of methods for depositing layers that differ in terms of economics and cost-effectiveness [4,5,6,7,12].

Among all the methods for obtaining nanocomposite materials that can be found in the literature, the electrodeposition method is a versatile and economical technique compared to other techniques [13,14]. Electrochemical deposition offers the possibility of obtaining new materials by codepositing solid particles suspended in an electrodeposition bath [4,5,6,7,13,14]. The electrodeposition technique is widely used to obtain nanocomposites with a metal matrix as it has a number of advantages, such as low cost, industrial applicability, uniform deposition, control of layer thickness, etc. [14].

Interest in this method has grown substantially because it is also an industrial procedure that is applied in order to produce layers that ensure corrosion and wear resistance, and it can also change the thermal, magnetic and optical characteristics of surfaces [4,5,6,7]. The literature shows that the factors that influence the process of obtaining nanocomposite layers are current density, pH, bath temperature, nanoparticle concentration, agitation and nanoparticle size [4,5,6,7,14]. Particles of Al_2_O_3_ [15,16], CeO_2_ [17,18], SiC [19,20], TiC [4,5,6,7,21,22,23], TiO_2_ [24] and ZrO_2_ have so far been electrodeposited in nickel matrixes [25,26], as well as WC [27] with dimensions between 10 nm and 10 µm. Regarding the existing data in the literature on the electrodeposition of titanium carbide nanoparticles (TiC), there are studies on the influence of electrodeposition parameters [4,5], on tribocorrosion resistance [5,7] and on the characterization of the obtained layers [6], but very little data about the corrosion resistance of these layers [28].

TiC is preferred as a reinforcing material in the nickel metallic matrix due to its high melting point, high mechanical rigidity, good corrosion and wear resistance, high hardness and low density [4,5,6,7]. Ni/TiC nanocomposites are thus good candidates for use in surface protection in nuclear power plants, the automotive and aerospace industries and other systems.

The novelty of this study consists in the knowledge added to the literature about the corrosion resistance of Ni/nano-TiC layers as compared to pure Ni layers, highlighting the effect of the nanosized TiC dispersed phase included in the nickel matrix during the electrodeposition process. This kind of information has not been published before.

The aim of this study was to comparatively characterize Ni/nano-TiC layers and pure Ni layers by using electrochemical methods to evaluate their corrosion resistance in specific environments involving an energy fields, such as nuclear power plants. The results of the electrochemical tests to monitor corrosion resistance showed that the inclusion of TiC nanoparticles in the Ni matrix had the effect of improving the corrosion resistance compared to pure electrodeposited Ni layers.

## 2. Results and Discussion

### 2.1. SEM Surface Morphology and Layer Thicknesses

**Figure 1:**
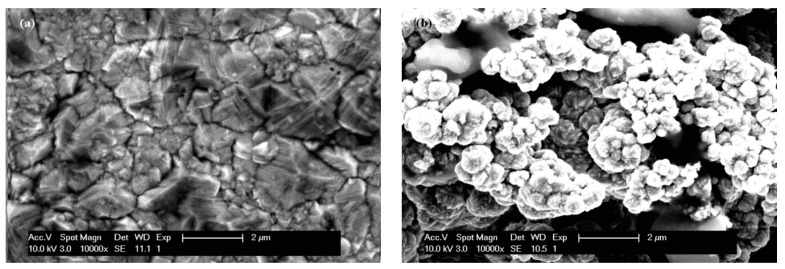
SEM surface micrographs of: (**a**) pure Ni layer; (**b**) Ni/nano-TiC composite layer.

Before evaluation of the corrosion resistance of the layers obtained, they were characterized with the help of an electronic microscope, both on the surface and in cross-section. Figure 1 shows the surface morphologies of the layers of pure nickel and the Ni/nano-TiC layers obtained with the same current densities and electrodeposition times.

As can be seen in Figure 1, the inclusion of TiC nanoparticles in the nickel matrix during the electro-codeposition process resulted in changes in the surface morphology of the nanocomposite layer compared to the pure nickel layer. Thus, the surface structure changed from the specific pyramidal electro-crystallization of pure nickel to the global, irregular, cauliflower-type structure with microcrystalline aggregates that can be seen on the surface of the Ni/nano-TiC nanocomposite layer [5,6].

**Figure 2:**
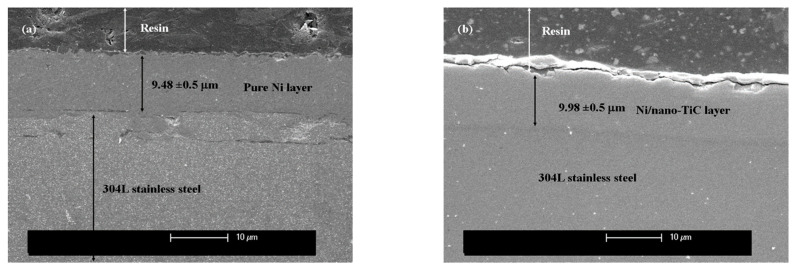
SEM micrographs of layer thickness for: (**a**) the pure Ni layer; (**b**) the Ni/nano-TiC composite layer.

The thickness of the obtained layers was estimated both by weighing before and after electrodeposition and by using SEM micrographs obtained for the samples processed in cross-section. The thickness was 9.48 µm for pure nickel and 9.98 µm for the Ni/nano-TiC composite layer. SEM micrographs of the layer thicknesses for pure nickel and the composite layer are shown in Figure 2.

As can be seen in Figure 2, the layers of pure nickel and of composite nickel doped with TiC nanoparticles were approximately the same thickness, were adherent to the substrate and had flawless continuity.

### 2.2. XRD Diffraction Patterns

**Figure 3:**
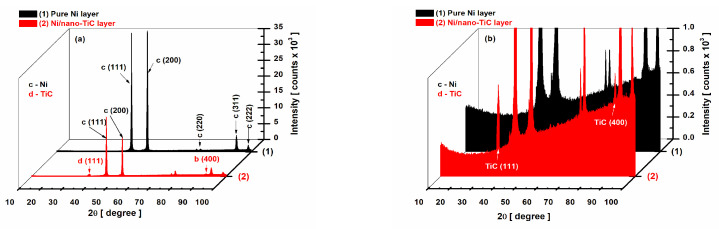
X-ray diffraction patterns for electrodeposited layers: (1) pure Ni layer; (2) Ni/nano-TiC composite layer. (**a**) Full intensity counts; (**b**) magnification of the scale in order to show the inclusion of TiC nanoparticles into the Ni matrix with the specific peaks.

Figure 3 compares the diffractograms obtained by XRD analysis for the pure nickel layer and the Ni/nano-TiC composite layers.

With the help of the Inorganic Crystal Structure Database (ICSD) program, the following crystallographic phases were identified with nine-digit codes.

For the pure Ni layer found in the program database with the code ICSD 01-071-3740, the Ni element belonging to the cubic crystallization system, the space group Fm-3m was identified with the planes (111), (200), (220), (311) and (222) at the diffraction angles 2θ of 44.48°, 51.84°, 76.37°, 92.92° and 98.42°. The peak intensities recorded were 36,573.84, 377,779.82, 913.18, 5036.85 and 1717.17 counts, respectively (Figure 3a, curve (1)).

For the Ni/nano-TiC composite layer (Figure 3a, curve (2)), it can be observed that the Ni element kept its crystallographic planes and diffraction angles 2θ as in the layer without the addition of nanoparticles, but with a reduction in the intensity of the peaks characteristic of the Ni matrix at 2θ angles of, respectively, 44.48°, 51.84°, 76.37°, 92.92° and 98.42°, the peaks having an intensities of 18,684.82, 12,521.84, 1595.04, 2577.80 and 1246.92 counts. In addition to the Ni element in the Ni/nano-TiC layer, there was also the scattered phase of the TiC, identified with the code ICSD 01-089-3828, belonging to the cubic crystallization system; the Fm-3m space group was identified at 2θ angles of 35.99° and 91.96° and the crystallographic planes (111) and (400), with peak intensities of 823.77 and 755.52, respectively.

As shown in Figure 3a, a decrease in the intensity of the peaks corresponding to pure nickel was observed with the addition of TiC nanoparticles in the nickel electrolyte and their incorporation in the nickel matrix. Compared to the intensity of the peaks from the pure nickel layer without the addition of TiC nanoparticles, the intensity of the peaks of the nickel element decreased; for example, for the plane (111), the intensity of the Ni element from the pure nickel layer at the angle 2θ = 44.48° was 377,779.82 counts, which decreased to 18,684.82 counts in the Ni/nano-TiC composite layer (Figure 3 curve (1) and curve (2)). This behavior can be attributed to the inclusion of TiC nanoparticles in the nickel matrix and the reduction in the size of the Ni crystals. While, at the pure Ni layer, we had an average crystal size of 132 ± 0.1 nm, at the Ni/nano-TiC layer, the average size of the Ni crystals from the nickel matrix was only 98 ± 0.1 nm.

For the two systems studied, the average crystal size was calculated with the Debye–Sherrer formula (1) [29]:

(1) $$d_{XRD}=\frac{0.9\lambda }{FWHM\cdot \cos\theta }$$

where d*_XRD_* is the crystalline size, *λ* is Cu K_α_ 1.54506 Ǻ, *FWHM* (in radians) is the full width at half-maximum of the characteristic peak and *θ* is the diffraction angle.

After including TiC nanoparticles in the nickel matrix through the electro-codeposition process, a decrease in Ni crystallites was observed with the addition of the dispersed phase concentration, thus demonstrating that TiC nanoparticles induce the nanostructuring of the nickel metallic matrix.

### 2.3. Evolution of Open Circuit Potential

**Figure 4:**
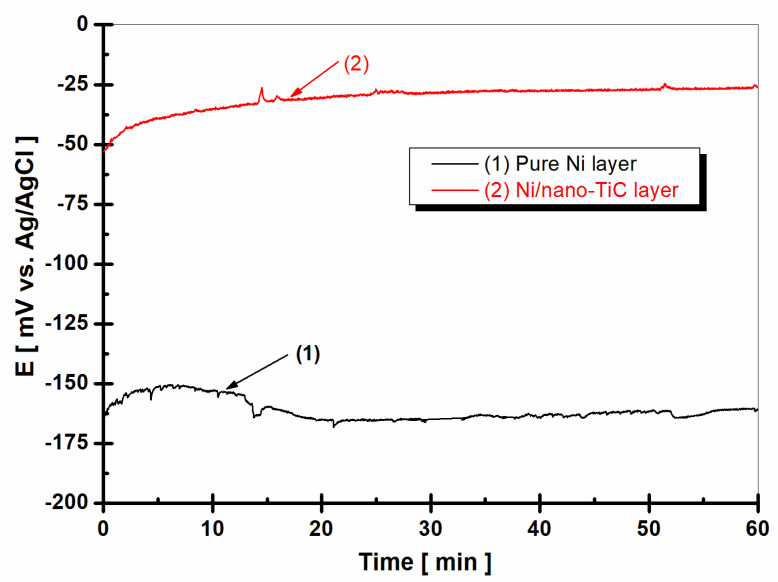
Evolution of the free potential (OCP) during 1 h of immersion in H_3_BO_3_-LiOH solution: (1) pure Ni layer; (2) Ni/nano-TiC layer.

The open circuit potential (OCP), or free potential, is the potential established between the working electrode and the electrochemical medium used to test the samples compared to that for a reference electrode, which is placed in the electrolyte close to the working electrode. The open circuit potential (OCP) method reveals the state of the surface of the studied material as a function of the immersion time in a solution and is a non-destructive method. This method provides information about the tendency of the tested material to oxidize or passivate in an electrochemical environment. Figure 4 shows the evolution of the free potential after 1 h of immersion in a boric water solution (LiOH-H_3_BO_3_), which simulated the water in the primary circuit of nuclear power plants, for the pure Ni layer compared to the Ni/nano-TiC layer composite layer.

From Figure 4, we can see that the free potential of the pure nickel layer (curve (1)) started from a value of about −160 mV vs. Ag/AgCl and decreased during the measurement time towards more negative values, down to the value of approximately −170 mV vs. Ag/AgCl. The tendency to move towards more negative values shows us that the surface of the tested material, in this case the pure nickel layer, was incapable of forming a protective oxide layer in the tested electrochemical environment, instead being active and therefore subject to the corrosion process.

In contrast to the pure nickel layer, the free potential (OCP) of the Ni/nano-TiC composite layer (Figure 4, curve (2)) began at a much more positive value of −50 mV vs. Ag/AgCl and had a tendency to move towards higher values, reaching the value of approximately −25 mV vs. Ag/AgCl at the end of the 60 min. The trend towards more positive values reveals that a protective oxide layer was more easily formed on the surface of the sample in which embedded TiC nanoparticles were present, thus producing a passive state in the composite layer, with a higher corrosion resistance compared to the pure Ni layer.

**Figure 5:**
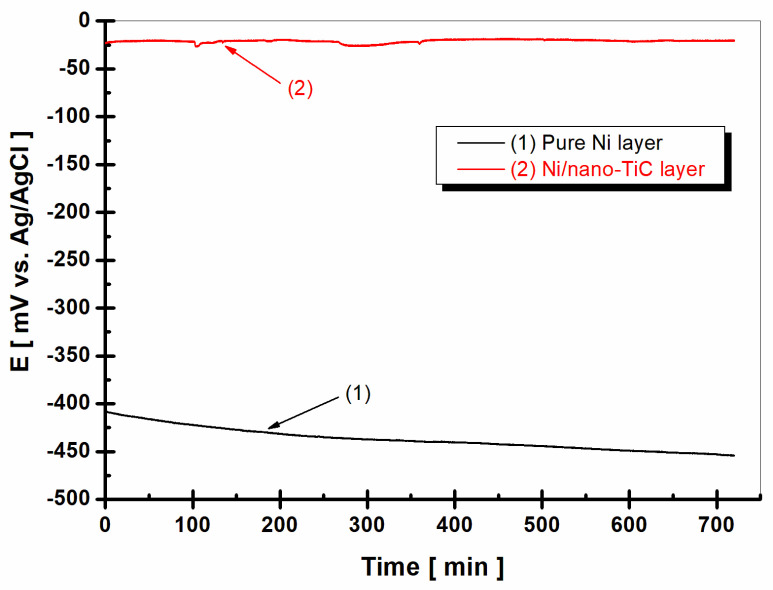
Evolution of the free potential (OCP) over 12 h after 36 h immersion in H_3_BO_3_-LiOH solution: (1) pure Ni layer; (2) Ni/nano-TiC layer.

After 36 h of immersion, (Figure 5, curve (2)), it was noticed that, for the Ni/nano-TiC layer, the value of the free potential remained constant after 60 min of immersion (around −25 mV vs. Ag/AgCl), demonstrating that, for the nanocomposite layer, a state of equilibrium was reached after approximately 55 min. In contrast, for the pure Ni layer (Figure 5, curve (1)), the value of the free potential (OCP) constantly moved towards more negative or more active values. While, after 60 min of immersion, the value of the potential was approximately −170 mV vs. Ag/AgCl, after 48 h of immersion, a decrease in the potential down to approximately −450 mV was noticed. The potential difference was 290 mV lower than the value of the free potential for the pure Ni layer at the end of the 60 min of immersion.

The evolution of the free potential demonstrated a beneficial influence from the incorporation of TiC nanoparticles into the nickel metallic matrix during the electrodeposition process, changing the free potential values to more positive (nobler) fields compared to the pure Ni layer, thus giving the Ni/nano-TiC composite layer a passive state that was more resistant to corrosion in the respective solution. The results obtained by monitoring the free potential were further supported by the results from measuring the electrochemical impedance.

### 2.4. Electrochemical Impedance Spectroscopy (EIS)

Electrochemical impedance spectroscopy (EIS) is a modern study method and the most complex method for determining the corrosion behavior of a material.

This method consists of disturbing the system with an alternating signal, superimposed on the direct current that normally supplies the electrochemical cell, and measuring the response. Thus, the cell can be connected to an alternating current bridge that allows the determination of the impedance [15,19,20,25,27]. The representation of the electrochemical impedance spectroscopy diagrams can be in Nyquist form or in the complex plane, where the real part, or Real Z, is represented on the abscissa and the imaginary part, or –ImZ, on the ordinate. In Bode representations, the impedance modulus or phase angle is represented on the ordinate and the frequency in logarithmic form is represented on the abscissa.

**Figure 6:**
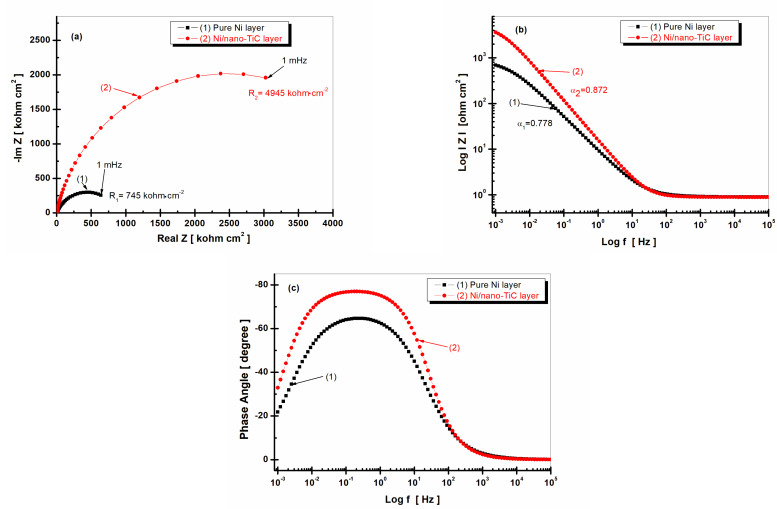
Electrochemical impedance spectroscopy results shown as symbols and the fitted line after 1 h immersion for (1) pure Ni layer; (2) Ni/nano-TiC layer. (**a**) Nyquist representation; (**b**) module Z versus logarithm of frequency; (**c**) phase angle versus logarithm of frequency.

Figure 6 shows the electrochemical impedance spectroscopy diagrams recorded after 1 h of immersion of the studied layers in the form of Nyquist (Figure 6a) and Bode (Figure 6b,c) plots.

**Figure 7:**
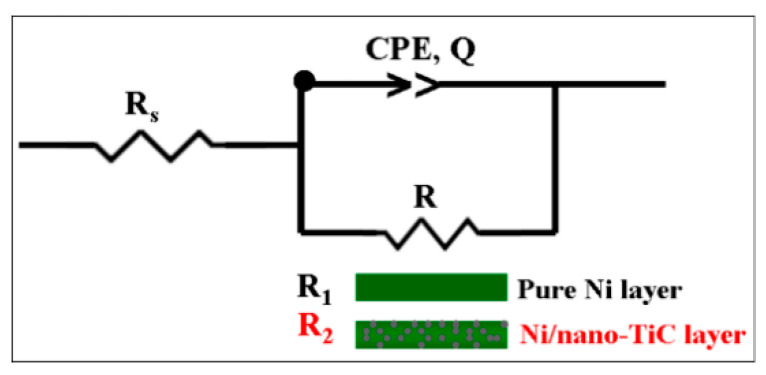
Equivalent electrical circuit representing the pure Ni and Ni/nano-TiC layers immersed in LiOH-H_3_BO_3_ solution.

In order to study and model the experimental data, Z_View_ software was used, and the quality of the experimental data and the fitted data were evaluated with the help of the chi-squared test, which has values lower than 10^−3^. For the simulation of the experimental data, an equivalent electrical circuit was used, composed of the solution resistance (R_s_); the specific resistance of the studied layer (respectively, R_1_ for the pure nickel layer and R_2_ for the Ni/nano-TiC composite layer); and constant phase elements (CPE_1_ and CPE_2_), which replaced the electric double-layer capacity for each system because the Nyquist diagrams did not look like perfect semicircles due to the roughness and inhomogeneity of the studied surfaces. The equivalent electrical circuit is shown schematically in Figure 7.

In Figure 7, R_s_ represents the resistance of the solution between the reference electrode and the working electrode, R_1_ is the specific resistance of the pure Ni layer, R_2_ is the specific resistance of the Ni/nano-TiC composite layer and CPE is a constant phase element, which was used to simulate impedance data when the Nyquist diagram was not a perfect semicircle, possibly due to the uneven and inhomogeneous surface of the electrode. The impedance of such an electrochemical system with a CPE is expressed by Equation (2) [30,31].

(2) $$Z_{CPE}=\frac{1}{Q{\left(j\omega \right)}^{\alpha }}$$

where the CPE parameter, *α,* is also known as the CPE exponent and *Q* is the magnitude of CPE measured in F cm^−2^s*^α^*^−1^; *j* is an imaginary number, *j* =$\sqrt{-1}$; ω is the angular frequency expressed in rad s^−1^; and *f* is the frequency in Hz (2π*f*).

The parameter α can take values from –1 to 1. When α ≠ 1, the system exhibits behavior that has been attributed to surface heterogeneity [22,23,24,25].

From Figure 6a, it can be observed that the specific resistance of the Ni/nano-TiC composite layer had a value of 4945 kohm cm^2^, which was about six times higher than the specific resistance of the pure Ni layer, which had a value of only 745 kohm cm^2^. This behavior confirmed the beneficial effect of the TiC nanoparticles embedded in the Ni matrix on the corrosion resistance of the composite layer in the solution, which simulated the water in the primary circuit of nuclear power plants. The impedance modulus shown in Figure 6b was also higher for the Ni/nano-TiC composite layer (curve (2)), with a value for the parameter α of 0.872, compared to the impedance modulus of the pure nickel layer, which had a smaller value for the parameter α of 0.778. The phase angle from Figure 6c was higher for the Ni/nano-TiC composite layer (curve (2)) compared to the phase angle recorded for the pure Ni layer (curve (1)). In addition, the phase angle of the composite layer maintained a constant value of approximately 78 degrees over a wider range of frequencies, indicating good stability for this layer in the studied solution.

**Figure 8:**
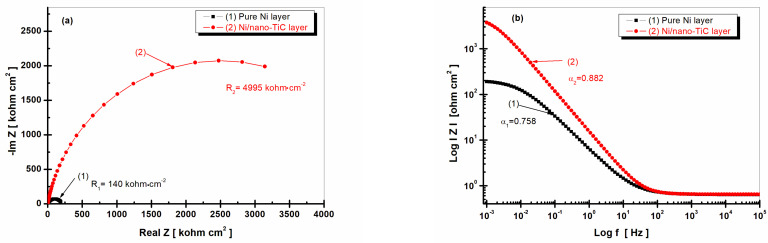

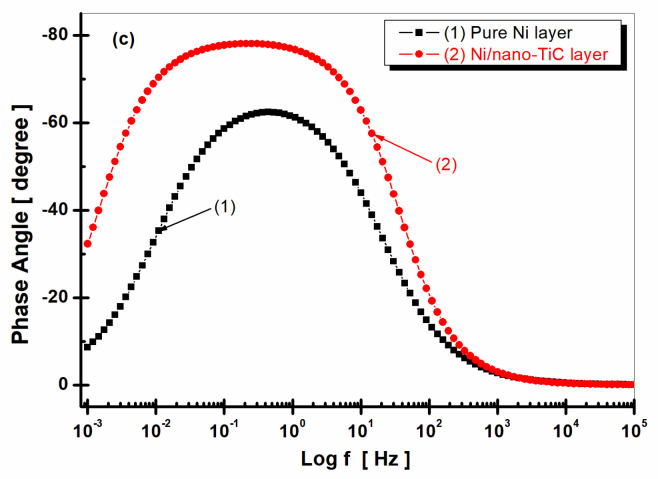
Electrochemical impedance spectroscopy results shown as symbols and fitted lines after 48 h of immersion for (1) the pure Ni layer; (2) the Ni/nano-TiC layer. (**a**) Nyquist representation; (**b**) module Z versus logarithm of frequency; (**c**) phase angle versus logarithm of frequency.

Figure 8 shows the EIS diagrams recorded after 48 h of immersion of the samples for the pure Ni layer and the Ni/nano-TiC layer.

From Figure 6a and Figure 8a, it can be observed that, for the pure Ni layer, the specific resistance decreased with the increase of the immersion time. While, after 1 h of immersion, the specific resistance had a value of 745 kohm cm^2^, after 48 h of immersion, it reached a lower value of 140 kohm cm^2^, resulting in a decrease of about 15-fold with increasing time of immersion. For the Ni/nano-TiC composite layer, the specific resistance increased slightly with increasing immersion time. While, after 1 h of immersion, the specific resistance of the Ni/nano-TiC layer had a value of R_2_ = 4945 kohm cm^2^, after 48 h, the R_2_ value increased slightly to 4995 kohm cm^2^, thus resulting in the difference between the specific resistance of the Ni/nano-TiC layer and the pure Ni layer after 48 h of immersion becoming approximately 35 times higher.

Figure 6b and Figure 8b, which represent the impedance modulus as a function of frequency, show that the slope of the transition region between the limits of the two asymptotes, indicating the power of the dependency of the imaginary part of the impedance on the frequency [30], was different for the two layers. The analysis of the slopes showed a higher value for the Ni/nano-TiC layer that slightly increased for both immersion periods. In the case of the Ni/nano-TiC layer, α increased from 0.872 to 0.882 (curve (2)) as compared to the pure Ni layer, which showed a decreasing value with the increase of the immersion period. The value of α was 0.778 after 1 h of immersion and decreased to 0.752 after 48 h of immersion (curve (1)).

The Bode diagrams representing the phase angle as a function of frequency (Figure 6c and Figure 8c), show a phase angle value closer to −80 degrees for the Ni/nano-TiC composite layer, which remained constant throughout the immersion period. This behavior indicates poor reactivity and better corrosion resistance in the composite layer that had nanoparticles of TiC in the nickel matrix compared to the pure Ni layers, which had a value close to −60 degrees that also remained constant but in a narrower range of frequencies.

The TiC nanoparticles embedded in the nickel metallic matrix did not take part in the charge transfer process during the corrosion process but acted as insulating centers on the composite surface and reinforced the passive film, therefore improving the corrosion resistance.

The results for the EIS measurements were in line with the results for the open circuit monitoring during the immersion period.

**Figure 9:**
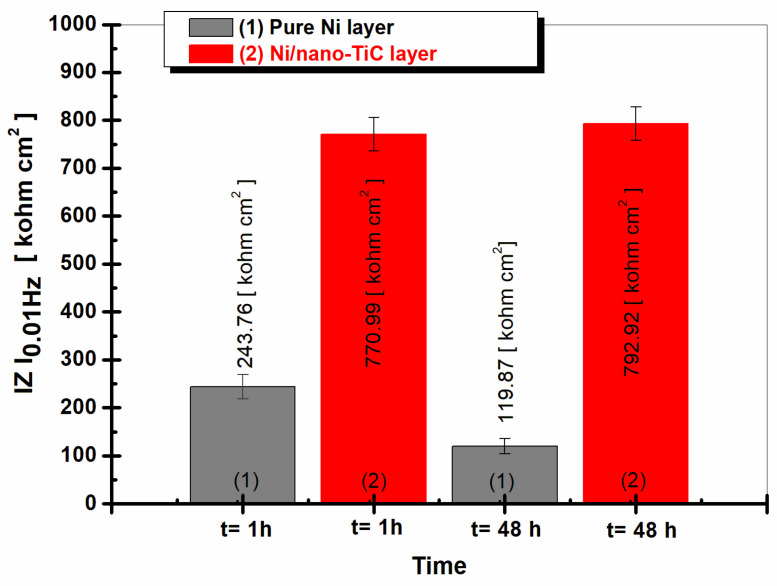
The low-frequency impedance modulus at 0.01 Hz obtained in LiOH-H_3_BO_3_ solution after 1 h and 48 h immersion for different samples: (1) pure Ni layer; (2) Ni/nano-TiC layer.

Figure 9 shows the values for the low frequency impedance modulus (Z_0.01 Hz_) for the pure Ni layer compared to the Ni/nano-TiC layer for the two different time periods (respectively, 1 h and 48 h of immersion).

At low frequencies, the values of the impedance modulus can be attributed to the processes that take place at the layer–electrolyte interface, where the corrosion phenomenon begins [32]; they can function as effective tools in elucidating the studied system to determine the corrosion resistance. The values of the low frequency impedance module (Figure 9) for Ni/nano-TiC were higher than for the pure Ni layers for both immersion times studied.

It was noticed that the Z_0.01 Hz_ value for the pure Ni electrodeposited layer after 1 h of immersion was 243.76 kohm cm^2^, while the Z_0.01 Hz_ value for the Ni/nano-TiC composite layer was higher by about three times at about 770.99 kohm cm^2^. After 48 h of immersion, the Z_0.01 Hz_ value for the pure Ni layer decreased to 119.87 kohm cm^2^, while the Z_0.01 Hz_ value for the Ni/nano-TiC composite layer increased slightly, being about six times higher than the corresponding Z_0.01 Hz_ value for the pure Ni layer at 792.92 kohm cm^2^.

Figure 9 also shows that the values Z_0.01 Hz_ for the pure Ni layer decreased with the increase of the immersion time. While, after one hour of immersion, it had a value of 243.76 kohm cm^2^, at 48 h of immersion, it reached the value of 119.87 kohm cm^2^. The layer in which nanoparticles of TiC were added showed a slight increasing value for Z_0.01 Hz_; after 1 h of immersion, the value was 770.99 kohm cm^2^, which increased slightly to 792.92 kohm cm^2^ after 48 h of immersion.

## 3. Materials and Methods

To obtain pure Ni and Ni/nano-TiC layers, a typical deposition electrolyte was used for the Ni deposition—namely, a Watts electrolyte—containing nickel sulfate and nickel chloride with a pH of 4.04 ± 1. The deposition was performed potentiostatically with an electrochemical workstation in a double-walled electrochemical cell consisting of three electrodes: the working electrode (WE), or cathode; the anode, or counter electrode (CE), consisting of a pure nickel plate; and the reference electrode. The electrolyte volume for deposition was 450 mL, the stirring regime was 450 rpm (established by testing for the best homogeneous dispersion of TiC nanoparticles) and a bath temperature of 45 ± 1 °C was used [4,5,6,7].

**Figure 10:**
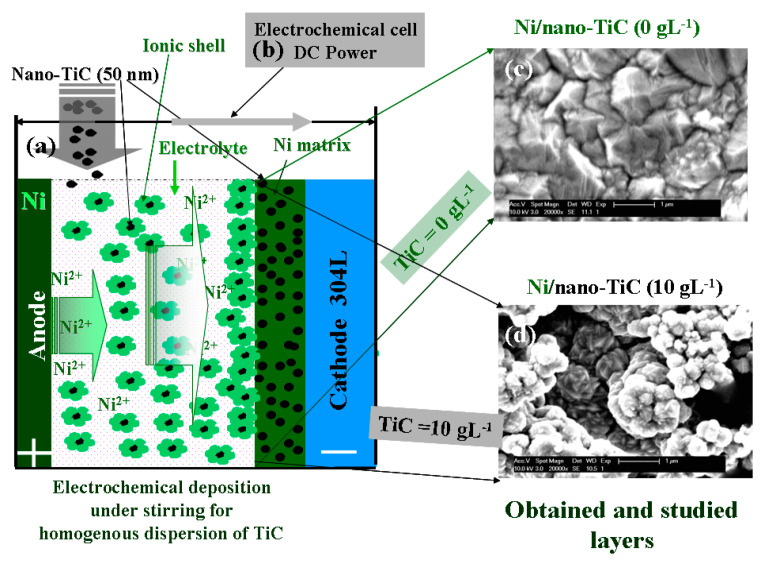
Schematic of the electro-codeposition process used to obtain Ni/nano-TiC nanocomposite layers: (**a**) electrochemical cell; (**b**) electrochemical workstation; (**c**) SEM surface micrograph of pure Ni layer; (**d**) SEM surface micrograph of Ni/nano-TiC layer.

The layers were obtained at a current density of 60 mA cm^−2^ and with a deposition time of 10 min. The concentration of TiC nanoparticles present in the plating electrolyte was 10 gL^−1^, thus resulting in a dispersed phase concentration for the TiC embedded into the nickel matrix of 3.86 wt%. The average diameter of the TiC particles was 50 nm. A schematic of the electrochemical cell for the electrodeposition of layers of pure nickel and nickel doped with TiC nanoparticles is presented in Figure 10.

A solid nickel plate with an active surface area of about 50 cm^2^ was used as a counter electrode or anode to keep the nickel ion concentration of the electrolyte constant. The reference electrode used was calomel (Hg/Hg_2_Cl_2_, saturated KCl), which functioned to measure the relative potential of the working electrode. The working electrode or support material on which the layers were electrodeposited was a 304 L stainless steel plate with a thickness of 2 mm and an active surface of 25 cm^2^, this being the cathode on which the nickel reduction reaction took place [4,5,6,7]. The chemical composition of 304 L stainless steel is shown in Table 1.

TiC nanopowder with an average nanoparticle size of 50 nm was purchased from Hefei Kaier Nanometer Energy & Technology Co. Ltd. (Anhui, China) [4,5,6,7]. The characteristics of the TiC nanopowder are presented in Table 2.

To evaluate the corrosion resistance, a solution consisting of a mixture of 5.72 gL^−1^ LiOH plus 0.4485 gL^−1^ boric acid dissolved in distilled water was used, which simulated the composition of the water in the primary circuit of nuclear power plants, the electrolyte having a pH = 8.3 [7].

An electrochemical workstation was used to characterize the corrosion behavior of the electrodeposited layers. The experimental setup used a double-walled electrochemical cell to maintain a constant temperature and a solution volume of 350 mL. The cell contained three electrodes: the working electrode (WE), which was formed of either electrodeposited pure Ni or Ni/nano-TiC layers, these being the samples compared; the counter electrode (CE), consisting of a platinum–rhodium grid; and the reference electrode composed of Ag/AgCl (with saturated KCl solution), which had a stable potential value of +199 mV compared to the normal electrode of hydrogen (NHE).

**Figure 11:**
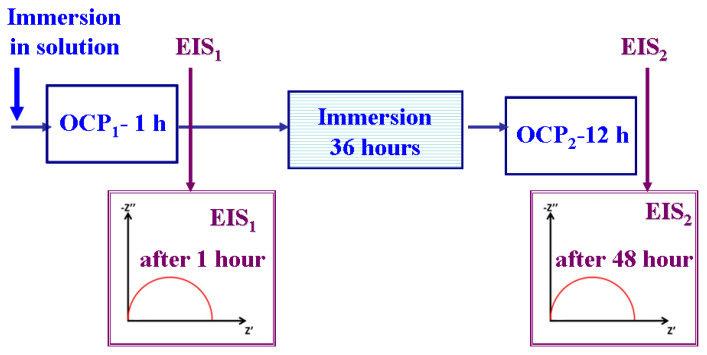
Schematic representation of the experimental protocol.

The electrochemical system was connected to a computer equipped with a program to control the experiment and record experimental data. Data analysis software was used to process the data and present diagrams. The analyzed samples were insulated with resin to obtain a well-defined, real active surface of 3.67 cm^2^. Before each experiment, since the samples were cleaned after the electrodeposition process, they were only washed with distilled water before immersion in the test solution. Corrosion tests were performed at an ambient temperature of 25 ± 1°C. The measurements were repeated four times to verify the reproducibility of the experimental data. A schematic representation of the experimental protocol for the evaluation of corrosion resistance is shown in Figure 11.

As can be seen in Figure 11, the experimental protocol for investigating the corrosion resistance of the obtained layers consisted of the following:

- Measuring the evolution of the free potential E(t), which represents the variation in time of the electrode potential (WE) in an open circuit (OCP), over 60 min.
- Plotting the electrochemical impedance spectroscopy (EIS) diagrams at free potential and in alternating current, with an amplitude of AC = 10 mV in the frequency range from 100 kHz to 1 mHz, with 20 frequencies per decade.
- Immersing the samples in solution for 36 h.
- Measuring the evolution of the free potential E (t), or the OCP, over 12 h.
- Plotting the electrochemical impedance spectroscopy (EIS) diagrams after 48 h of immersion.

A Philips XL 30FEG electron microscope was used for the morphological characterization of the surfaces and the thickness of the obtained layers. For the analysis of the thickness of the layers in cross-section, the samples were embedded in a special resin.

To identify the crystalline phases, their preferred orientation and the comparative sizes of the nickel crystallites in the pure nickel layers and in the composite layers, X-ray diffraction (XRD) analyses were performed on the obtained surfaces. The equipment used for XRD analysis was a Seifert 3003 T diffractometer. The diffractograms were recorded with a copper cathode at a voltage of 40 kV and an intensity of 40 mA, with a step of 0.021°/s when varying the angle 2θ from 10° to 100°.

## 4. Conclusions

This research highlighted the beneficial effect of TiC nanoparticles embedded in a Ni matrix during the electrodeposition process on the corrosion resistance of a Ni/nano-TiC nanocomposite layer in a solution that simulated water from the primary circuit of nuclear power plants. Corrosion resistance was evaluated using electrochemical methods.

From the surface morphological analysis of the electrodeposited layers, a modification of the layer morphologies was identified with the inclusion of TiC nanoparticles in the Ni metal matrix. The Ni/nano-TiC layer had a cauliflower-like morphology, while the pure Ni layer had the pyramidal crystals specific to the electro-crystallization of nickel.

From the morphological analysis of the cross-section, a slight increase in the thickness of the Ni/nano–TiC layer was observed compared to the pure nickel layer obtained with the same time and current density.

In the evolution of the free potential, there was a shift in the potential towards more positive values with the addition of TiC nanoparticles in the Ni metallic matrix and with the increase of the immersion time compared to the pure Ni layer. The tendency to move towards more noble potentials indicates that a protective oxide film with a higher corrosion resistance was more easily formed on the surface of the Ni/nano-TiC layer compared to the pure Ni layer without the addition of nanoparticles.

Electrochemical impedance spectroscopy diagrams showed an increase in the specific resistance with increasing immersion time for the Ni/nano–TiC composite layer compared to the pure Ni layer. At the same time, for the pure Ni layer, the specific polarization resistance decreased with increasing immersion time.

After 48 h of immersion, the specific resistance for the Ni/nano-TiC composite layer had a value that was about 35 times higher than the specific resistance of the pure Ni layer.

The values of the impedance modulus (Z_0.01Hz_), extracted from the results for the electrochemical impedance spectroscopy at low frequency, confirmed the beneficial effect of adding TiC nanoparticles into a nickel matrix to improve the anticorrosive performances of the layers obtained from the electrochemical deposition compared to the layer without added nanoparticles.

TiC nanoparticles embedded and dispersed into a nickel matrix during the electrodeposition process induce the nanostructuration of the metallic matrix by decreasing the crystallite size.

This study brings new information about the effect of TiC nanoparticles embedded in a Ni metallic matrix on the improvement of corrosion resistance, which may be useful in successfully utilizing these layers in the energy field.

## Figures and Tables

**Table 1:** Chemical composition of 304 L stainless steel.

Chemical Composition (%)									
C	Si	Mn	P	S	Ni	Cr	N	Cu	Mo
0.013	0.490	1.140	0.031	0.006	8.030	18.21	0.027	0.240	0.140

**Table 2:** The characteristics of TiC nanopowder.

Characteristics	Technical Specifications
Color	Black
Morphology	Powder
Purity	>99.0%
Crystalline structure	Cubic
The specific size of the surface	23 m^2^/g
Particle dimension	50 nm
Density at 20 °C	4.93 g/cm^3^
Melting point	3050–3230 °C
Boiling point	4800 °C

## Data Availability

Not applicable.
